# Predicting the Patriarchal Politics of Pandemics From Mary Shelley to COVID-19

**DOI:** 10.3389/fsoc.2021.624909

**Published:** 2021-03-24

**Authors:** Eileen Hunt Botting

**Affiliations:** Department of Political Science, College of Arts and Letters, University of Notre Dame, Notre Dame, IN, United States

**Keywords:** Mary Shelley, Octavia Butler, Margaret Atwood, patriarchy and masculinity, populism and democracy, COVID-19, dystopian literature, women's rights

## Abstract

I examine the predictive powers of the political science fictions of Mary Shelley, Octavia Butler, and Margaret Atwood for understanding the patriarchal—or men-dominant—dynamics of the politics of pandemics in the twenty-first century. Like her literary followers in post-apocalyptic plague literature, Butler and Atwood, Shelley foresaw that the twenty-first century would be the age of lethal pandemics. Their post-apocalyptic fictions also projected the ways that patriarchal and authoritarian forms of populism could shape the cultural circumstances that can turn a local outbreak of a new and deadly contagious disease, like COVID-19, into a politically chaotic and economically devastating global plague. Modern feminist political science fiction born of Shelley's great pandemic novel *The Last Man* (1826) is seemingly clairvoyant not because of any supernatural powers of the authors but rather because of their studied attention to the wisdom of plague literature, the lessons of epidemic history, and the political dynamics of patriarchy and populism.

## Introduction

Because it anticipates some of the “dystopian” or “horrifying” elements of the 2020 U.S. presidential election year (Shames and Atchison, 2019, p. 7), Mary Shelley's 1826 pandemic novel *The Last Man* provides an illuminating theoretical lens on the perils of patriarchal and authoritarian forms of populism in modern democracies during times of wider epidemiological crisis (Washington, 2019, p. 89; Coffee, p. 2020). Set in the late twenty-first century, her third novel after *Frankenstein* (1818) has been one of the most influential, yet still unsung, works of modern “political science fiction” (Hassler and Wilcox, 1997, Botting, 2020a, p. 1–29). Its narrative premise of a global plague that seemingly leaves one human male survivor has since influenced countless works of fiction, film, and television (Botting, 2020a, p. 30–45). Like *Frankenstein* before it, *The Last Man* is best understood as a “political science fiction” because it analyzes the political causes, consequences, and implications of human technological, scientific, or other artificial or cultural change of their natural, social, and built environments (Botting, 2020a, p. 1–29). Shelley vividly illustrated the political basis and exacerbation of pandemics by picturing the origins of a mass extinction event on the “pestilential battlefields” of a centuries-long war between Greece and Turkey (Botting, 2021, p. 36).

*The Last Man* creatively employs the tropes of ancient epic fiction from Homer to Ovid, yet sets them in the future, in order to transform the modern novel into a medium for conducting the probabilistic political science of pandemics. Her novel is an extended narrative thought experiment that hypothesizes the wartime political origins, and projects the likely social and economic consequences of plagues—whether they are narrowly conceived as biological contagions or more broadly understood as disastrous concatenations of human conflicts and failures in response to the spread of infectious or contagious diseases. In so doing she created a template for modern feminist political science fiction writers, such as Octavia Butler and Margaret Atwood, who have used plague as a setting and a metaphor for theorizing the deeper political pathologies of human societies—past, present, and future (Atwood, 1986, 2004, 2009, 2013; Butler, 2012).

## The Definition of Patriarchy, Authoritarianism, and Populism Within Modern Feminist Literature

Like Butler and Atwood after her, Shelley thought that women would be hurt more than men by the diseased state of democracies in times of epidemic and medical crisis. And the reason would be what the radical feminist political theorist Catharine MacKinnon calls the “systematic” (or overt) and “systemic” (or implicit) privileging of men in positions of power—usually white, Western, and wealthy—over women, disempowered men, children, people of color, immigrants, and other political minorities who comparatively lack formal and informal means of exercising control over the government agenda (MacKinnon, 2006, p. 29). More recently, the feminist philosopher Kate Manne (2020, p. 3) has focused her critical attention on conceptualizing the enduring sense of “entitlement of privileged men,” and explaining how such male privilege accumulates in cultural and political institutions over time to produce the material and legal conditions for women's subordination and inequality relative to men.

As John Stuart Mill argued in his groundbreaking feminist treatise *The Subjection of Women* (Mill, 1869), patriarchal societies tend to lend credence to the unfounded opinion that some people have greater authority over others simply by virtue of their masculine gender and other arbitrary and artificial senses of social and economic privilege. Patriarchal societies hence beget conditions for authoritarian political rule, or top-down modes of governance whereby a single leader or body of representatives—even if democratically elected—can impose their will upon the ruled through either the threat or the actual use of force. Authoritarian forms of government are thus opposed to democratic and liberal societies in which the equal rights and freedoms of the citizenry are put first and foremost among the constitutional standards that are secured by the rule of law (Rawls, 1999, p. 14).

As part of a modern tradition of women's rights literature that dates to the work of Mary Wollstonecraft, Shelley's mother, feminist political science fictions help us to assess how patriarchal and authoritarian forms of populism bring harm and injustice to women and other disempowered people. While “populism” is presently understood as an “essentially contested” concept, social scientists have articulated a minimalist definition of its three main features (Mudde and Kaltwasser, 2017, p. 3, 5). Populism is a style and set of strategies in politics that focuses on “attacking ‘the elite’; defending the interests of ‘the common people’; and proclaiming ‘popular sovereignty as the only legitimate source of political power”’ (Kantola and Lombardo, 2019, p. 1108).

Through rhetoric, iconography, leadership, and other political activities, populism tends to posit that a moral and cultural homogeneity defines the character of the corrupt elite class as well as its opposite, the virtuous body of the people, whose collective will challenges the illegitimacy of the elite's power. Populists invoke the notion of the purity and unison of the will or voice of the people, and seek to impose its values upon the polity for the sake of the good of the whole. Although populists typically uphold themselves as defenders of the sovereignty of the people against would-be tyrants, they simultaneously use the idea of the homogenous and pure will of the people to battle dissent and repress pluralism of values in the wider liberal or democratic society that they seek to reform, control, or overturn (Müller, 2016, p. 41–49). These three basic features of populism—anti-elitism, defense of the common people, and belief in the foundational value of popular sovereignty—appear across many of its historical varieties, on both the left and the right sides of the political spectrum (Mudde and Kaltwasser, 2017, p. 3, 5). However, social and political scientists still disagree as to whether any general definition of populism is sufficient to capture its phenomena in all their complexities, including their differentiation by gender, or right-wing or left-wing ideologies (Kantola and Lombardo, 2019, p. 1108).

Populism's forms are as diffuse as its present conceptual definition. It encompasses varieties of social movements, democratic electoral politics, party systems, and executive leadership that invoke the moral authority of the ordinary people over and against the alleged illegitimacy of elite political actors in order to ascend to either democratic governance or authoritarian rule of the people whom they purport to speak for (Müller, 2016, p. 41–49). Paradoxically, populist movements often appeal to gender, sexual, racial, disability, class, and national stereotypes in fostering distrust of democratic political institutions, or politics altogether, even as they profess to support the empowerment of “the people” (Ackerly, 2020; Botting, 2020b).

A similar yet wider dynamic animates patriarchal politics. Even as it strives to empower an elite class of men, patriarchal politics exploits and manipulates stereotypes of masculinity, femininity, race, class, (dis)ability, (hetero)sexuality, and other social statuses to publicly intimidate and demean women and other (including male) political minorities in a way that makes them appear or feel subordinate to those same privileged (often white and wealthy) men. Patriarchal strains of populism do not only mobilize men to be strident, aggressive, and even violent supporters of radical political causes, whether they are on the left or the right wing of the spectrum, but also perversely convert women to become models and advocates of their condescending view that women are meant to be servants of men, not equal rights-bearing citizens alongside them (Love, 2020; Tiffany, 2020; Bracewell, 2021).

Recent feminist social science scholarship—empirical and normative—has begun to build a fourth dimension of the growing consensus on the definition of populism. This fourth dimension of the definition of populism runs parallel to the insights of the political science fictions of Shelley, Butler, and Atwood. This is the view that populism poses distinct challenges for feminist politics and the realization of women's rights, women's democratic representation, and women's leadership (Abi-Hassan, 2017; Kantola and Lombardo, 2019; Ackerly, 2020; Hirschmann, 2020; Mostov, 2021). Mostov (2021) has contended that “populism is always gendered and dangerous for women and democracy” because of the ways that populist rhetoric, leadership, and political activity draws on gendered, racialized, classed, and nationalistic ideas to separate people into the opposing camps of “the elite” and “the people,” a blunt distinction that both encourages and relies upon crude ideological line-drawing that isolates and marginalizes vulnerable groups in society. Like Hirschmann (2020), Mostov distinguishes between populism (whether on the left or the right) and those leftist “popular movements,” such as the civil rights movement in the United States, which seek reform of society and laws without trying to overturn foundational democratic ideals and institutions of legitimate governance, such as the protection of equal rights and freedoms for each and every citizen.

Even when women (whether white or people of color, rich or poor) are agents, leaders, or short-term or selfish beneficiaries of it, populism poses both systemic and systematic dangers to all political minorities precisely because it seeks to manipulate society's gendered, racialized, classed, ableist, and nationalized expectations of them in the name of realizing the supposedly homogenous and pure will of the people (Love, 2020; Bracewell, 2021; Mostov, 2021). During the political and economic instability of the COVID-19 pandemic, patriarchal, and authoritarian forms of populism—most vividly and effectively led by the right-wing Republican U.S. President Donald Trump (Honig, 2018)—have taken advantage of the chaos to both target and control women and other political minorities, in different ways, while simultaneously undermining public “enjoyment” of equal civil rights and liberties guaranteed under the U.S. Constitution and reinforced by international human rights standards (Ackerly, 2016, p. 26). By returning to the epic yet futuristic storyline of Shelley's *The Last Man*, we can learn to step back and see the potential trajectory of the patriarchal, authoritarian, and populist politics of a global pandemic, and perhaps anticipate and avert these destructive patterns of political behavior as we look ahead.

## How Shelley's *The Last Man* Reveals the Patriarchal Politics of Populism

Shelley predicted that even the wealthiest and most powerful of modern democracies can fail in the face of the political strains and fissures revealed in its “institutions” by the economic and social stressors of a cataclysmic pandemic (Coffee, 2020). Indeed, *The Last Man* even projected that the world's leading republic of her time, the United States, would not exist by the year 2092: in its place would be the “Northern States of America,” a different regime pushed to the brink of anarchy by a global plague (Shelley, 2006, Vol. II, Chap. 6). Her seminal post-apocalyptic novel identified three patterns of modern democratic corruption, which would be exposed and exacerbated by a pandemic: (1) slow yet steady institutional erosion of norms and practices of trust and equality, (2) authoritarian forms of populism that betray the people who bring an executive leader to power, and (3) patriarchal and religious forms of populism that manipulate the people's beliefs through fear and disinformation. These three forms of democratic corruption map onto the three-volume narrative structure of the political novel itself.

In volume I, Shelley charts how decades of political corruption precede the visitation of the most devastating wave of plague to England. Until recently a monarchy, England is a new and fragile democratic regime or representative republic. Despite the appearance of the free and fair election of Lord Raymond to the executive position of Lord Protector, democratic institutions begin to erode due to the moral corrosion of the elite white men in power who drive and manipulate the mechanisms of government behind the scenes (Shelley, 2006, Vol. I, Chap. 6). When Lord Raymond selfishly abdicates his position to escape an ill-fated love affair by going to war in Constantinople in defense of the independence of Greece, the populist leader, Ryland, takes advantage of the resultant chaos to ascend through electioneering to the position of Lord Protector. Ryland eventually abdicates the position, too, leaving the former prince turned republican, Adrian, to step in as the interim deputy Lord Protector just as the plague arrives in London—but it is too late for even a true defender of liberty to save democracy from falling apart^1^.

It is in volume II that the professed man of the people, Ryland, ascends to the highest executive office in the land. Although he claims to have the interests of the “popular party” at heart, he dismantles the egalitarian system of social welfare put in place by his predecessor Lord Raymond (Shelley, 2006, Vol. I, Chap. 4). Although the state has the technology and infrastructure to distribute food and other necessities to all of its citizens, it conspicuously fails to do so as the plague seeps into its most dense and populous city (Shelley, 2006, Vol. I, Chap. 7; Vol. II., Chap. 6). In an ironic turn of events, the populist executive gets infected by the airborne pathogen. Ryland shows his true selfish character by renouncing responsibility for the people who elected him. But when he is found dead, “half-devoured by insects,” alone in a quarantine bunker far from the capitol, it was not the final political tragedy to befall the twenty-first-century republic (Shelley, 2006, Vol. III, Chap. 1). The collapse of the democratic political system, and the decimation of its population by the plague, had yet to come.

In volume III, a new and dangerous populist leader arises from the rubble of the representative republic. This time he is a charismatic and authoritarian Christian preacher and “patriarch” (Shelley, 2006, Vol. III, Chap. 5). The itinerant “methodist” evangelizes and manipulates the vulnerable remnant of plague survivors—targeting women, the poor, and children—to seek health and protection in his apocalyptic cult (Shelley, 2006, Vol. III, Chaps. 5–6). His medical disinformation and religious propaganda make his followers naively believe that they are immune from the stealthy and invisible transmission of the deadly disease. As the “plague, slow-footed, but sure in her noiseless advance” besets even the “elect” of the cult, the patriarch lies about the numbers of the infected and even hides the bodies of his own followers in a desperate attempt to cement his prophetic authority among his chosen people (Shelley, 2006, Vol. III, Chap. 26).

In outlining these patterns of democratic backsliding, patriarchal populism, and rising authoritarianism in the wake of a pandemic, Shelley paved the way for a new strain of feminist post-apocalyptic literature. As we shall see, the novels of Butler and Atwood follow *The Last Man* in imagining political regimes where war, corruption, authoritarianism, patriarchy, and populism work together to propel the leveling spread of a deadly pathogen. But before we can fully appreciate the theoretical import of their counterfactual twenty-first-century futures, we need to touch down in the political present to see how dystopia has played out in the real world for women during the novel coronavirus pandemic.

## Are We (Women) Living in a Plague Dystopia Now?

Since the COVID-19 quarantines, lockdowns, and mitigation policies began in early 2020, people have been asking themselves, “Are we living in a dystopia?” (Atchison and Shames, 2020). Political scientists Shauna Shames and Amy Atchison define a “dystopia” as a fictional place, as imagined in literature, film, and other arts, which serves as a warning of the authoritarian corruption of popular “civil liberties” in societies and governments that had once treated their citizens as equally free and responsible under the limits of the law (Shames and Atchison, 2019, p. 10). They distinguish between these fictional “dystopias,” which serve as warnings of what not to do in order to avoid democratic backsliding into authoritarianism, and those “dystopian” societies and governments which in fact use authoritarian or antidemocratic tactics (including “totalitarian” or all-controlling policy measures) to “oppress” the people (Shames and Atchison, 2019, p. 21–23).

Since she wrote her post-apocalyptic bioengineered plague trilogy set in the near twenty-first-century future, the *MaddAddam* series (2003–2013), Atwood has advanced a similar view about the relationship between fictional dystopias and dystopian societies (Atwood, 2010). It is intriguing to see the most prominent contemporary feminist author of political science fiction converge with political scientists on two key points: (1) dystopias are fictional but have roots and parallels in the history and contemporary journalism of real-world “dystopian” politics, and (2) they imagine “planned,” “patriarchal,” “authoritarian,” and “totalitarian” regimes in order to reasonably guess, if not exactly “predict,” patterns of democratic backsliding in the future, especially for those people most vulnerable to oppression in the present (Atwood, 2019, Appendix on her Archives; Atchison and Shames, 2020; Atwood, 2020).

During the state-mandated lockdowns last spring, Shames, Atchison, and Atwood almost simultaneously argued that people are not in fact living in plague dystopias. In their shared view, the early emergency and temporary COVID-19 mitigation measures taken by governments around the world last spring—no matter how demanding or extreme—did not in fact erode civil liberties and human rights but rather protected the lives and safety of people during an unprecedented global health crisis. But as the pandemic wore on, leaving over a million deaths in its wake, dystopia felt more like a clear and present threat (World Health Organization, 2020). The United States stood on the cusp of either the re-election of Donald Trump or his active resistance to a peaceful transfer of power to the legitimate winner (Fisher, 2020). As we reflect back on the tumultuous events of January 2021—the storming of the capitol by racist insurrectionists, followed by the unprecedented second impeachment of Donald Trump for inciting the seditious mob violence, and the heavily guarded inauguration of Joe Biden as the 46th president of the United States—it would perhaps be safer to say that Americans are not living under a dystopian government *yet*.

The existential and political question of whether we are living in a plague dystopia has perhaps never been more poignant for women around the world. Since the economic lockdowns and school and daycare shutdowns began in February 2020, women have disproportionately faced threats to their physical safety at home and at work, beginning with spikes in domestic violence and extending to their service on the front lines of the health care and service industries (Doctors without Borders, 2020). Many women—from the U.S. to Poland—have effectively lost access to reproductive and other gender-specific health care, or have been denied the right to elect abortion in their home states or countries (Baker, 2020; Magdziarz and Santora, 2020). Thousands of women lost their jobs during the economic lockdowns or gave up their careers to care for their children due to the competing pressures of online learning and working from home (Schmidt, 2020). Women have likewise seen a decline in affordable, safe, and quality childcare and educational systems for their families (Taub, 2020).

At the same time, the tragedies and injustices of the pandemic have produced a cosmic feminist irony. Countries with a history of supporting the political empowerment of women have done better at mitigating the spread of COVID-19 (Wigley and Wigley, 2020). The gendered political paradox is clear. While women-empowering democratic countries such as New Zealand have succeeded in flattening the curve of the spread of COVID-19 through adherence to strict public health protocols, women in men-dominant regimes—including democracies like the U.S.—have faced rapid backsliding in their empowerment and equality as citizens, parents, and economic actors.

Given these countervailing trends, it would seem that some women are in fact living under dystopian governments while dealing with the effects of the COVID-19 pandemic. Others live in more utopian, democratic, women-empowering societies that have proven to be better at protecting not only public health but also the basic equal rights and liberties of citizens regardless of gender or other social status. The comparative analysis of the plague novels of Shelley, Butler, and Atwood can help us to see how dystopian governments and societies tend to be gendered in a way that is patriarchal, or privileging of powerful men, in their broader behavioral and legal patterns of democratic backsliding and rising authoritarianism. These women writers' political science fictions also unmask how the patriarchal and authoritarian aspects of populism foster conditions for women's oppression and other forms of manipulation of vulnerable or marginalized people during times of wider medical crisis such as a pandemic.

## Patriarchy, Populism, and Pandemics in Contemporary Feminist Political Science Fiction

After the model of Mary Shelley's *The Last Man* (1826), Octavia Butler's *Clay's Ark* (1984) and *Parable of the Talents* (1998), and Margaret Atwood's *The Handmaid's Tale* (1985) and *MaddAddam Trilogy* (2003-13) made a series of uncanny predictions about how patriarchal forms of populism would shape the dystopian politics of pandemics in the twenty-first century. We have seen how the political story of *The Last Man* depicted the toxic convergence of democratic backsliding, patriarchal and evangelical forms of populism, and pandemic-induced economic and electoral chaos to cause the toppling of the world's most powerful representative republic. Although all three writers used the twenty-first century as the futuristic backdrop for their post-apocalyptic plague novels, it was Butler who presciently chose the year “2021” as the precise date for the man-made disasters of *Clay's Ark* (Butler, 2012, p. 41).

Clay's Ark is a crashed spaceship with one male survivor—a “Last Man” of sorts—who unleashes an extraterrestrial microorganism onto the Earth. The infected astronaut Eli turns other humans and their offspring into cat-like, non-human predators. They mate with those they inoculate to increase their numbers as well as to promote the rapid spread of the spores of the contagion. Led by the charismatic Eli who colonizes their ranch in the desert, the infected establish a cult-like society in which they forcibly impress fertile young human women to serve as wives and mothers for the reproduction of the new species.

Butler's political science fiction even seems to have anticipated the powerful strain of patriarchal populism led by Donald Trump. In *Parable of the Talents*, she described the rise of a religious populist patriarch in the 2030s United States who pledges to his followers to “Make America Great Again” (Aguirre, 2017). Scholars agree that Butler wrote in the science fiction and horror tradition of Shelley's *Frankenstein* (Goss and Riquelme, 2007). I would add that Butler must have also taken inspiration from *The Last Man*, especially Shelley's gothic vision of the “imposter-prophet” who wants to be “remembered by the post-pestilential race as a patriarch, a prophet, nay a deity” (Shelley, 2006, Vol. III, Chap. 5). This “methodist” or evangelizing Christian preacher (Shelley, 2006, Vol. III, Chap. 5) leads the apocalyptic plague cult that falsely promises immunity to members in return for their religious worship of his growing authoritarian political power (Washington, 2019, p. 89; Coffee, 2020).^2^ Butler may have taken special inspiration from Shelley's tragic depiction of one of the women pushed into the cult in crafting the character of Rane in *Clay's Ark*. Revealing his malevolent quest for power, the false prophet stabs and kills the innocent Juliet and strangles her baby in front of his remaining followers who have not yet fallen to the plague (Shelley, 2006, Vol. III, Chap. 6). In a parallel turn of horrific misfortune, the teenage Rane faces brutal rape and decapitation after her capture by a cult-like “car family” that preys on other people lost on the desert frontier of an American society rapidly backsliding into anarchy (Butler, 2012, p. 358).

A reader of the post-apocalyptic fiction of Shelley and Butler, Atwood composed her *MaddAddam* series—*Oryx and Crake* (2003), *The Year of the Flood* (2009), and *MaddAddam* (2013)—with many of the structural elements and themes of their futuristic plague novels. Putting an explicitly feminist twist on the “Last Man” narratives of Shelley and Butler, *The Year of the Flood* takes place in the immediate aftermath of a bioengineered plague (made by a misanthropic, young male scientist reminiscent of Victor Frankenstein) that seems to have wiped out all but two female human survivors (Atwood, 2013, p. 28). When they were young and even more vulnerable, without means of supporting themselves independently in their sexually violent, male-dominated, and increasingly anarchistic society, Toby and Ren were forced to join a green, back-to-the-Earth, apocalyptic plague cult, founded by a charismatic patriarch named “Adam One” (Atwood, 2009, p. 48). Although Adam One originally imposes a strict vegan lifestyle upon his followers, he ultimately uses his self-made (and self-aggrandizing) religion to justify cannibalism of his own tribe—even a religious obligation to “sacrifice our own protein”—when the plague that he prophesied arrives and brings the survivors to the edge of starvation (Atwood, 2009, p. 413–415).

The predicament of the women in *The Year of the Flood* recalls the harrowing story of Atwood's most famous feminist political science fiction. *The Handmaid's Tale* unfolds the story of Offred, a woman who has been forced to serve as a sexual surrogate (or handmaid) for a military commander and his wife. The white supremacist theocratic regime, Gilead, arose after a fundamentalist Christian terrorist organization staged a successful coup of the northeastern United States in the wake of a series of “epidemic” crises of sexually transmitted disease and toxic contamination of the environment that caused widespread depopulation, infertility, and birth defects (Atwood, 1986, p. 303–304). A segregated society, Gilead purges black and gay people, and sends dissidents (mainly women) to work in colonies where they die from exposure to toxic waste and pollution. Taking place around the year 2005, *The Handmaid's Tale* provides a prescient commentary on twenty-first-century America's ongoing political failure to care for people of color and protect their rights to life and health. Akin to laborers in Gilead's colonies, Black, Hispanic, and Latinx people are 2.8 times more likely to die after contracting COVID-19 than their white counterparts in the U.S., in large part because of underlying health conditions derived from their experience of economic and political inequality (CDC, 2020).

Dressed in red robes and white puritan hats, the still-fertile handmaids of Gilead have neither rights over their own bodies nor rights to parent the children they bear for the patriarchs who rape them under the watchful eyes of their wives. Since the 2016 election of Donald Trump, feminist activists around the world have donned handmaid costumes to protest the 45^th^ American president's public history of misogynist prejudice and sexual harassment of women, and his administration's attempts to use its influence in the Republican Senate and the conservative Supreme Court to precipitate backsliding on women's civil and human rights, especially to reproductive freedom and heath care (Hauser, 2017). As if the partisan counter-script was also ripped straight from the pages of *The Handmaid's Tale*, the coronavirus-infected President Trump used and championed “remdesivir,” an antiviral drug made by the “Gilead” corporation, just after the September release of the results of World Health Organization-sponsored human trials demonstrated that it had no effect on either the recovery or the mortality of COVID-19 patients (Cohen and Kupferschmidt, 2020).

Atwood, Butler, and Shelley's works of modern feminist political science fiction are seemingly “clairvoyant” (Atwood, 2010)—not because of any supernatural powers of the authors, but rather because of their studied attention to the wisdom of plague literature of the past, the lessons of epidemic history, and the political dynamics of patriarchy and populism. Before she wrote *The Last Man*, Shelley was aware of a tradition of plague literature that extended from Thucydides' influential historical account of the plague of Athens to Daniel Defoe's historical novel about the Great Plague of London, *A Journal of the Plague Year* (1722), which was based on his uncle's diary of surviving the visitation of the bubonic plague to the city in 1665–66 (Shelley, 1987, Vol. I, p. 131, 137). Butler and Atwood also regularly allude to the modern literature of plagues and pandemics that stems from *The Last Man*, from Edgar Allan Poe's 1843 short story “The Masque of the Red Death” to Richard Matheson's 1954 novel *I Am Legend* (Butler, 2012, p. 90–97; Atwood, 2004, p. 326).

The expressly historical bases for their political science fictions gave these women writers the ostensibly prophetic ability to make probable conjectures about gendered patterns of politics that have proven to be remarkably accurate for our plague year of 2020 to 2021. Shelley, Butler, and Atwood share a special concern for women, and the negative impacts of patriarchy and populism upon their freedom and happiness, especially in times of health or environmental crisis. Their woman-centered interpretive lenses led them to theorize the political interconnections between male domination, democratic backsliding, rising authoritarianism, and human-exacerbated epidemics of poor health, mass death, and other vicious social contagions.

Although we have observed some of these disturbing patterns unfold during the COVID-19 pandemic, we should follow Atwood, Atchison, and Shames in remembering that fictional dystopias serve as a “warning” of what to avoid if we want to preserve democracy and equal civil rights and liberties for each and all (Atchison and Shames, 2020; Atwood, 2020). By looking back to the political science fictions of Shelley, Butler, and Atwood, we gain a historical perspective on what needs defending and protecting in order to prevent further backsliding into destructive patterns of patriarchy, authoritarianism, and democratic corruption. From these forward-looking fictions, we also learn to identify the signs of incipient forms of patriarchal populism and how to effectively critique them in public.

The many women who have marched on Washington D.C. and New York City in the red and white costumes of Atwood's handmaids, protesting the conservative jurisprudence of Justice Barrett and the patriarchal populism of the President Trump, brought feminist activism back to its literary roots in our plague year of 2020 to 2021 (Fisher, 2020). The powerful allegories of modern feminist political science fiction have fast become a public code and index for tracking and resisting the breakdown of both democracy and the rights of women. What remains to be done in future scholarship on the sociology of gender, sex, and sexuality is to bring these and other works of feminist political science fiction into deeper conversation with the historical and empirical study of the dynamics of democracy, patriarchy, populism, and pandemics. If the current U.S. President Joe Biden is correct to say in his 2021 inaugural address that we must work to end “this uncivil war” that plagued American democracy under the right-wing populism of Trump, then it is high time to recognize the danger of our divisive “words” dealt in the political realm (Armitage, 2017, p. 67, 162–163; Biden, 2021). Sharp binary constructions of “us” vs. “them” only generate and exacerbate a cascade of divisions—between people of color and whites, poor and rich, rural and urban, plebes and elites, women and men, conservative and liberal—that threaten the very possibility of democracy, ironically, in the purported name of “the people” (Mostov, 2021).

## Author Contributions

The author confirms being the sole contributor of this work and has approved it for publication.

## Conflict of Interest

The author declares that the research was conducted in the absence of any commercial or financial relationships that could be construed as a potential conflict of interest.

## Footnotes

^1^*The Last Man* is a *roman à clef* in which Adrian is an avatar for Percy Bysshe Shelley, Mary Shelley's husband—the leading British Romantic poet of republican ideals. The narrator, and titular “Last Man,” Lionel Verney is the analog for Mary Shelley, who recorded in her journal in May 1824 that she felt like the “last man” of the second generation of English Romantic writers after the deaths of her husband in 1822 and their friend Lord Byron in the spring of 1824 (Shelley 1987, p. 55).

^2^Shelley's imposter-prophet may have been a commentary on an evangelical American itinerant methodist minister, Lorenzo Dow, or those like him in early nineteenth-century Ireland and England. Dow appealed to the Book of Revelation on the title page of his sermon *A Journey from Babylon to Jerusalem* (1812), calling for “my people” to escape “her sins”—“lest ye be partakers of her plagues” (Dow, 1812).
